# Interpretable machine learning-driven QSAR modeling for coagulation factor X inhibitors: from molecular descriptors to predictive potency

**DOI:** 10.1007/s10822-025-00758-2

**Published:** 2026-01-23

**Authors:** Ali Onur Kaya

**Affiliations:** Radiotherapy Department, Vocational School of Health Service, Akdeniz University, 07070 Antalya, Turkey

**Keywords:** Coagulation factor X, QSAR modeling, Machine learning, ExtraTreesRegressor, XGBoostClassifier, SHAP analysis, Applicability domain, Molecular descriptors, pKi prediction, Anticoagulant drug discovery

## Abstract

Inhibition of Coagulation Factor X (FXa) is a clinically validated therapeutic strategy; however, developing safer and more selective inhibitors remains a major challenge. In this study, we developed an interpretable machine learning–based QSAR framework to predict both the inhibitory potency and activity class of small molecules targeting FXa. A structurally curated dataset of 6400 compounds was retrieved from ChEMBL, standardized, and encoded using 391 non-redundant Mordred descriptors following systematic filtering. Benchmarking of 42 regression and 42 classification algorithms identified ExtraTreesRegressor and XGBoostClassifier as the most robust models. The regression model achieved an R^2^ of 0.760 and an RMSE of 0.831 on the independent test set, while the classification model reached an accuracy of 0.91 with balanced precision, recall, and an ROC-AUC of 0.962. SHAP (SHapley Additive exPlanations) analysis further enhanced interpretability by revealing that electrostatic, topological, and polar surface descriptors were the dominant contributors to FXa inhibitory potency. Applicability domain assessment using Williams plots confirmed that most compounds in both the training and test sets lay within the model’s reliable prediction space. Overall, the proposed QSAR pipeline integrates strong predictive performance with valuable mechanistic interpretability and rigorous validation, offering a practical computational tool for the virtual screening and rational design of novel FXa inhibitors.

## Introduction

Coagulation Factor X (FXa) is a vitamin K–dependent serine protease that bridges the intrinsic and extrinsic coagulation pathways and catalyzes the conversion of prothrombin to thrombin, making it a central regulator of fibrin clot formation [1–3]. Owing to this pivotal enzymatic role, FXa is a clinically important therapeutic target for the prevention and treatment of thromboembolic disorders, including deep vein thrombosis, pulmonary embolism, and atrial fibrillation–related stroke [4, 5]. Although direct oral anticoagulants (DOACs) such as rivaroxaban and apixaban have improved patient outcomes due to their predictable pharmacokinetics and reduced monitoring requirements, several clinical challenges remain unresolved. Persistent bleeding risks—particularly gastrointestinal and intracranial hemorrhage—along with interindividual variability in elderly patients, individuals with renal impairment, and those undergoing polypharmacy, highlight the need for next-generation FXa inhibitors with improved selectivity and safety profiles [6–8]. In this context, computational modeling approaches capable of rapidly exploring large chemical spaces can greatly accelerate the identification and optimization of novel anticoagulant candidates.

Over the past two decades, computational chemistry and ligand-based drug design have significantly contributed to understanding FXa–ligand interactions. Early 3D-QSAR studies provided foundational insights into the steric and electrostatic determinants of FXa inhibition [2, 8], while subsequent QSAR and machine learning (ML) approaches—including support vector machines (SVM), genetic algorithm–multilinear regression (GA-MLR), random forest, and gradient boosting—have demonstrated improved predictive capabilities by capturing nonlinear structure–activity relationships [3, 5, 9–12]. Despite these advances, several limitations persist. Many studies rely on relatively small or chemically narrow datasets, restricting their ability to generalize across broader chemical landscapes. Additionally, most existing models employ either regression or classification alone, limiting their capacity to provide complementary insights into potency and activity. A further challenge is the lack of descriptor-level interpretability, as many ML-based QSAR models operate as “black boxes,” offering limited mechanistic insights. Finally, the absence of rigorous applicability domain (AD) assessments reduces the reliability and practical utility of these models in virtual screening applications.

Recent developments in explainable artificial intelligence (XAI), particularly SHapley Additive exPlanations (SHAP), present promising opportunities to address these limitations by enabling transparent, feature-level interpretation of complex ML models [13–18]. When combined with large-scale data curation, algorithmic benchmarking, and AD analysis, interpretable ML-driven QSAR frameworks can deliver models that are both predictive and scientifically meaningful, offering a stronger basis for informed medicinal chemistry decisions [19–21].

Motivated by these scientific and methodological gaps, the present study introduces a comprehensive and interpretable ML-enhanced QSAR workflow for FXa-inhibitor discovery. Using a large and chemically diverse dataset of 6400 molecules curated from ChEMBL [22], the framework integrates regression models (ExtraTreesRegressor) for continuous pKi prediction and classification models (XGBoostClassifier) for active/inactive discrimination [23]. The inclusion of SHAP analysis provides mechanistic insights into the molecular features driving FXa inhibitory potency [16, 17], while Williams plot–based AD evaluation ensures prediction reliability across the chemical space [24]. By combining extensive data curation, dual-model predictive design, benchmarking-driven algorithm selection, mechanistic interpretability, and domain-aware validation, this study offers a transparent, generalizable, and scientifically informative computational foundation for the virtual screening and rational design of next-generation FXa inhibitors [19–21].

## Materials and methods

This section outlines the data curation workflow, descriptor generation and filtering strategy, model evaluation metrics, applicability domain assessment, and benchmarking-based algorithm selection used to develop the regression and classification models.

### Data collection and preprocessing

Bioactivity data for small-molecule Coagulation Factor X (FXa) inhibitors were retrieved from the ChEMBL database (Target ID: CHEMBL244) using the chembl_webresource_client Python API. Records reporting experimentally measured inhibitory constants (Ki) or half-maximal inhibitory concentrations (IC₅₀) were retained to ensure high-quality potency information suitable for QSAR modeling [3, 9]. The initial query yielded 8403 entries, including chemical structures and associated assay metadata.

Duplicate molecules were identified via canonical SMILES and removed to retain a single representative structure for each compound [3]. Activity values reported in nanomolar units were converted to a negative base-10 logarithmic scale using:

1 $$ p\;Activity = - \log_{10} \left( {stan dard \,value \times 10^{ - 9} } \right) $$

For consistency, this unified endpoint is referred to as pKi throughout the manuscript. This transformation produces a normalized potency scale correlated with binding free energy and is widely used in ligand-based modeling [3, 9].

Chemical structures were standardized, salts and counterions were removed, and compounds were filtered according to Lipinski’s Rule of Five (MW ≤ 500 Da, logP ≤ 5, HBD ≤ 5, HBA ≤ 10) to focus on drug-like molecules [11].

Following preprocessing, two task-specific datasets were constructed:

- *Regression dataset:* all 6400 molecules with continuous pKi values (range: 2.25–11.0).
- *Classification dataset:* molecules with intermediate potency (6.0 ≤ pKi ≤ 7.5) were excluded to avoid label ambiguity. The remaining 4645 compounds were labeled as active (pKi > 7.5; n = 2320) or inactive (pKi < 6.0; n = 2325), yielding a balanced dataset [3, 10].

### Descriptor calculation and feature filtering

Molecular descriptors were computed using the Mordred package, which provides a comprehensive library of 2D and 3D descriptor families, including topological, constitutional, geometrical, electrostatic, and physicochemical features. Canonical SMILES were converted into RDKit molecular objects and processed in Mordred to generate the initial descriptor matrix.

A structured, multistage filtering workflow was applied to ensure descriptor quality and reduce redundancy. First, descriptors with zero variance or more than 20% missing values were removed. Second, highly correlated descriptors (|r|> 0.80) were excluded. Third, multicollinearity was addressed by eliminating descriptors with a Variance Inflation Factor (VIF) greater than 5. Molecules for which descriptor computation failed due to structural instability were also discarded. After these preprocessing steps, 391 non-redundant descriptors were retained and used as the final feature matrix.

#### Descriptor selection strategy

To ensure a transparent and reproducible feature-selection process, an additional multiphase strategy was implemented. Zero-variance and high-missing-value descriptors were removed, followed by correlation-based filtering (|r|> 0.80) and VIF-based multicollinearity reduction (VIF > 5). Finally, feature importance derived from the ExtraTreesRegressor algorithm was used to prioritize predictive descriptors, and SHAP-based interpretability was applied to confirm their mechanistic relevance. This systematic pipeline yielded a stable, interpretable descriptor set for both regression and classification models.

### Model evaluation metrics

To evaluate the performance of the developed models, standard regression and classification metrics were applied.

For regression:

2 $$ R^{2} = 1 - \frac{{\sum (y_{i} - y_{i}^{\prime} )^{2} }}{{\sum (y_{i} - y^{\prime\prime})^{2} }} $$

3 $$ RMSE = \sqrt {\frac{{\sum (y_{i} - y_{i}^{\prime} )^{2} }}{n}} $$

where $y_{i}$. is the experimental pKi, $y_{i}{\prime}$ is the predicted value, and $y^{\prime\prime}$ is the mean of observed values. R^2^ measures the explained variance, wreas RMSE quantifies the average prediction error.

For classification: The accuracy, Precision, Recall, F1-score, and ROC-AUC were calculated using a confusion matrix [14].

4 $$ Accuracy = \frac{TP + TN}{{TP + TN + FP + FN}} $$

5 $$ Precision = \frac{TP}{{TP + FP}} $$

6 $$ Recall = \frac{TP}{{TP + FN}} $$

7 $$ F1 = 2 \times \frac{Precision \times Recall}{{Precision + Recall}} $$

where TP, TN, FP, and FN denote true positives, true negatives, false positives, and false negatives, respectively.

#### Dataset dplits

- Regression: 80% training (n = 5120), 20% test (n = 1280)
- Classification: 80% training and 20% test after removing the intermediate compounds.

### Applicability domain calculation

The applicability domain (AD) was assessed using the leverage approach and visualized using a Williams plot. The leverage values were computed as follows:

8 $$ h_{i} = x_{i}^{T} (X^{T} X)^{ - 1} x_{i} $$

with threshold:

9 $$ h^{*} = 3\frac{{\left( {p + 1} \right)}}{n} $$

where $p$ is descriptor count and $n$ the number of training molecules. Points with ${h}_{i}>{h}^{*}$ or standardized residuals outside ± 3 were considered outliers [13, 15].

This approach enables the evaluation of structural influence and error magnitude, ensuring that the predictions are interpreted within reliable chemical space boundaries [16].

SHapley Additive exPlanations (SHAP) analysis was applied to quantify the contributions of the descriptors and provide mechanistic insights into FXa inhibition.

### Benchmarking and model selection

To objectively determine the most suitable algorithms, a comprehensive benchmarking analysis was conducted using LazyPredict for regression and LazyClassifier for classification. A total of **42 regression** and **42 classification** models were evaluated under identical preprocessing steps and data splits.

Regression models were ranked using R^2^ and RMSE, while classification models were evaluated based on Accuracy, Precision, Recall, F1-score, and ROC-AUC.

The benchmarking consistently identified the ExtraTreesRegressor as the top-performing regression model and the XGBoostClassifier as the highest-performing classification model. This benchmark-driven approach ensured unbiased, reproducible, and reliable model selection.

## Results and discussion

In this section, the predictive performance, robustness, and interpretability of the regression and classification models are evaluated using benchmarking analyses, model-specific performance assessments, applicability domain examinations, and SHAP-based mechanistic interpretations.

### Benchmarking of regression and classification algorithms

To ensure transparent, unbiased, and reproducible model selection, a comprehensive benchmarking analysis was performed using the *regression and classification frameworks of LazyPredict*. A total of 42 regression and 42 classification algorithms were evaluated using identical data splits, descriptor sets, preprocessing procedures, and performance metrics. This large-scale benchmark allowed for the systematic identification of the most reliable algorithms for modeling FXa inhibitory potency and activity.

### Regression benchmarking

All regression algorithms were assessed using the independent test set R^2^ and RMSE values. The ensemble-based ExtraTreesRegressor delivered the strongest overall performance, achieving the highest R^2^ (0.760) and one of the lowest RMSE values (0.831). Additional ensemble learners, including RandomForestRegressor and GradientBoostingRegressor, also ranked among the top-performing models, whereas classical linear algorithms (e.g., Lasso, Ridge, and ElasticNet) consistently underperformed.

The strong performance of ensemble tree-based models can be attributed to the nonlinear nature of FXa–ligand interactions, which involve complex electrostatic, topological, and steric dependencies within the FXa binding pockets [25]. Linear models are generally unable to capture such higher-order feature relationships, whereas randomized decision tree ensembles excel in modeling heterogeneous chemical landscapes. This trend is consistent with recent FXa QSAR and ANN-QSAR studies demonstrating that nonlinear or ensemble learning methods often outperform linear models, particularly when analyzing structurally diverse inhibitor datasets [26].

Table 1 summarizes the top ten regression algorithms ranked according to their predictive performance. The observed ranking pattern confirms that FXa inhibition exhibits inherently nonlinear structure–activity relationships, reinforcing the suitability of ensemble learning for QSAR modeling in anticoagulant drug discovery research.

**Table 1:** Top 10 regression algorithms ranked by R^2^ and RMSE

Rank	Model	R^2^ (Test)	RMSE (Test)
1	ExtraTreesRegressor	0.760	0.831
2	RandomForestRegressor	0.742	0.856
3	GradientBoostingRegressor	0.721	0.892
4	XGBoostRegressor	0.708	0.915
5	LightGBMRegressor	0.702	0.926
6	AdaBoostRegressor	0.685	0.948
7	BaggingRegressor	0.671	0.962
8	KNeighborsRegressor	0.648	1.012
9	SVR (RBF kernel)	0.622	1.058
10	ElasticNet	0.598	1.093

#### Classification benchmarking

For the classification task, all 42 algorithms were evaluated using a comprehensive set of performance indicators, including accuracy, precision, recall, F1-score, and ROC-AUC. Among the evaluated models, the XGBoostClassifier exhibited the most consistent and superior performance across all metrics. Its balanced precision and recall values demonstrated a strong discriminatory power without favoring either the active or inactive class. Ensemble-based classifiers, such as RandomForestClassifier and LightGBMClassifier, closely followed, showing similarly robust performance.

The consistently strong performance of XGBoost and related ensemble methods can be attributed to the complex nonlinear decision boundaries that distinguish active from inactive FXa inhibitors (2). Subtle modifications in heterocyclic scaffolds, electronic substituents, or molecular polarity are known to cause substantial shifts in FXa inhibitory activity profiles [27]. In contrast, gradient-boosting algorithms such as XGBoost leverage sequential tree optimization, regularization, and feature-interaction learning, which makes them well-suited for chemically diverse QSAR datasets.

These benchmarking results are in line with earlier computational studies reporting that nonlinear machine-learning approaches frequently exhibit superior performance compared to classical statistical methods [19]. Overall, the benchmark results confirmed that boosting- and bagging-based ensemble models provided the highest predictive reliability for FXa activity modeling. The top ten classification models are listed in Table 2.

**Table 2:** Top 10 classification algorithms ranked by accuracy, F1-score, and ROC-AUC

Rank	Model	Accuracy	Precision	Recall	F1-score	ROC-AUC
1	XGBoostClassifier	0.91	0.92/0.89	0.89/0.92	0.91	0.962
2	RandomForestClassifier	0.89	0.90	0.88	0.89	0.945
3	LightGBMClassifier	0.88	0.87	0.88	0.88	0.941
4	GradientBoostingClassifier	0.88	0.89	0.87	0.88	0.936
5	ExtraTreesClassifier	0.87	0.88	0.86	0.87	0.930
6	SVC (RBF kernel)	0.85	0.86	0.85	0.85	0.912
7	LogisticRegression	0.83	0.84	0.83	0.83	0.901
8	KNeighborsClassifier	0.81	0.82	0.80	0.81	0.884
9	AdaBoostClassifier	0.79	0.80	0.78	0.79	0.870
10	NaiveBayes	0.76	0.77	0.76	0.76	0.855

### Regression model performance

A regression framework designed to predict the inhibitory potency (pKi) of Coagulation Factor X inhibitors was developed using a curated dataset of 6400 molecules encoded by 391 non-redundant Mordred descriptors. Following descriptor preprocessing and normalization, the dataset was divided into an 80% training subset and a 20% independent test subset to ensure unbiased model evaluation.

Among all regression algorithms assessed, the ExtraTreesRegressor demonstrated the strongest predictive performance, achieving an R^2^ of 0.760 and an RMSE of 0.831 on the independent test set. These metrics indicate that the selected descriptor set—capturing electrostatic, topological, steric, and physicochemical properties—effectively reflects key structural determinants of FXa inhibitory potency.

The strong performance of ExtraTreesRegressor is consistent with the multifactorial and nonlinear nature of FXa–ligand interactions. FXa binding involves contributions from charged residues in the S1 pocket, hydrophobic interactions within the S2 region, and aromatic stacking interactions in the S4 subsite, resulting in complex structure–activity patterns that are better captured by nonlinear models [25]. Furthermore, FXa inhibitors frequently feature diverse heterocyclic scaffolds and polar substituents, generating activity trends unsuitable for linear modeling frameworks.

Nonlinear machine-learning approaches have previously been shown to outperform classical regression methods when modeling FXa inhibitory activity, particularly for chemically heterogeneous libraries [9]. More recent in-silico anticoagulant studies incorporating ANN-QSAR and hybrid ML workflows similarly highlight the importance of nonlinear algorithms for capturing high-dimensional SAR patterns in FXa inhibitor datasets [26]

**Fig. 1:**
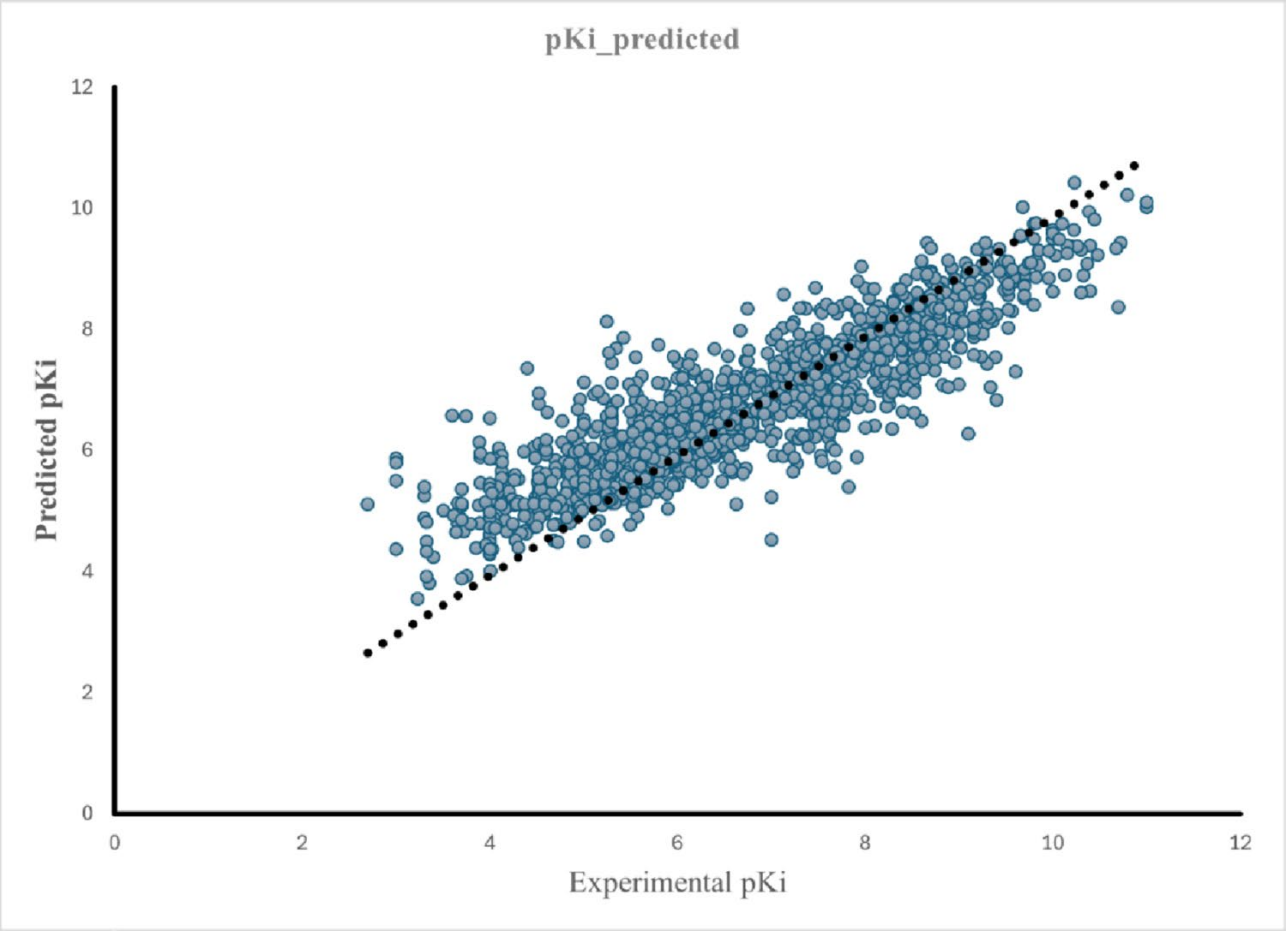
illustrates the correlation between the experimental and predicted pKi values for the test set, confirming the model’s consistent performance across chemical spaces.

Overall, the regression analysis demonstrates that ensemble-based learning provides a robust and reliable foundation for predicting FXa inhibitory potency across diverse chemical scaffolds, reinforcing the suitability of nonlinear ML models for virtual screening and rational design of next-generation FXa inhibitors (Fig. 1).

### Classification model performance

A binary classification model was developed to distinguish active (pKi > 7.5) from inactive (pKi < 6.0) Coagulation Factor Xa (FXa) inhibitors using the same curated descriptor matrix employed in the regression task. Molecules with intermediate potency values (6.0 ≤ pKi ≤ 7.5) were excluded to reduce class ambiguity and ensure a chemically meaningful separation between active and inactive compounds. This filtering strategy produced a balanced dataset of 4645 molecules (2320 active and 2325 inactive), encoded using 391 non-redundant Mordred descriptors capturing topological, electrostatic, steric, and physicochemical features. Stratified sampling was used to partition the dataset into an 80% training set and a 20% independent test set, preserving class distribution across both subsets.

A comprehensive benchmark involving 42 classification algorithms was conducted using the LazyClassifier framework. Consistent with the observations from the regression task, ensemble learning algorithms dominated the top rankings. Among them, the XGBoostClassifier demonstrated the highest and most balanced predictive performance. On the independent test set, the model achieved an accuracy of 0.91, with precision values of 0.92 (inactive) and 0.89 (active), recall values of 0.89 (inactive) and 0.92 (active), and an overall F1-score of 0.91. These metrics indicate that the classifier effectively captured key structural and electronic factors governing FXa inhibition without introducing systematic bias toward either class.

**Fig. 2:**
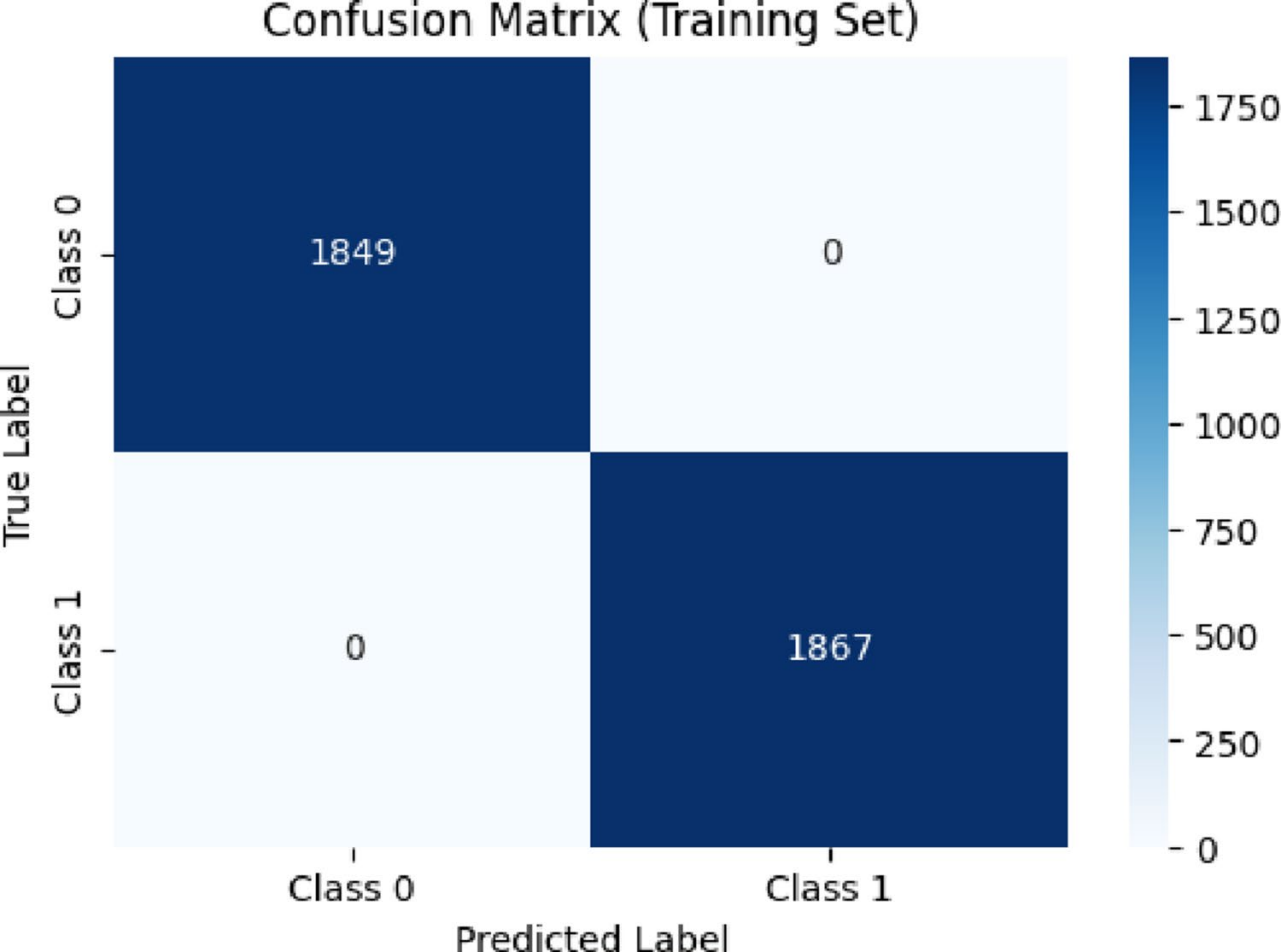
Confusion matrix of the XGBoost model for the training dataset

The confusion matrix for the training set (Fig. 2) showed complete class separation, with all 1849 inactive and 1867 active compounds correctly classified. While this outcome reflects strong learning capacity, such flawless internal performance may also indicate the possibility of overfitting—particularly in chemically diverse datasets where gradient-boosting models can memorize subtle descriptor patterns. Thus, independent test-set evaluation is essential to confirm meaningful generalization rather than training-set memorization.

**Fig. 3:**
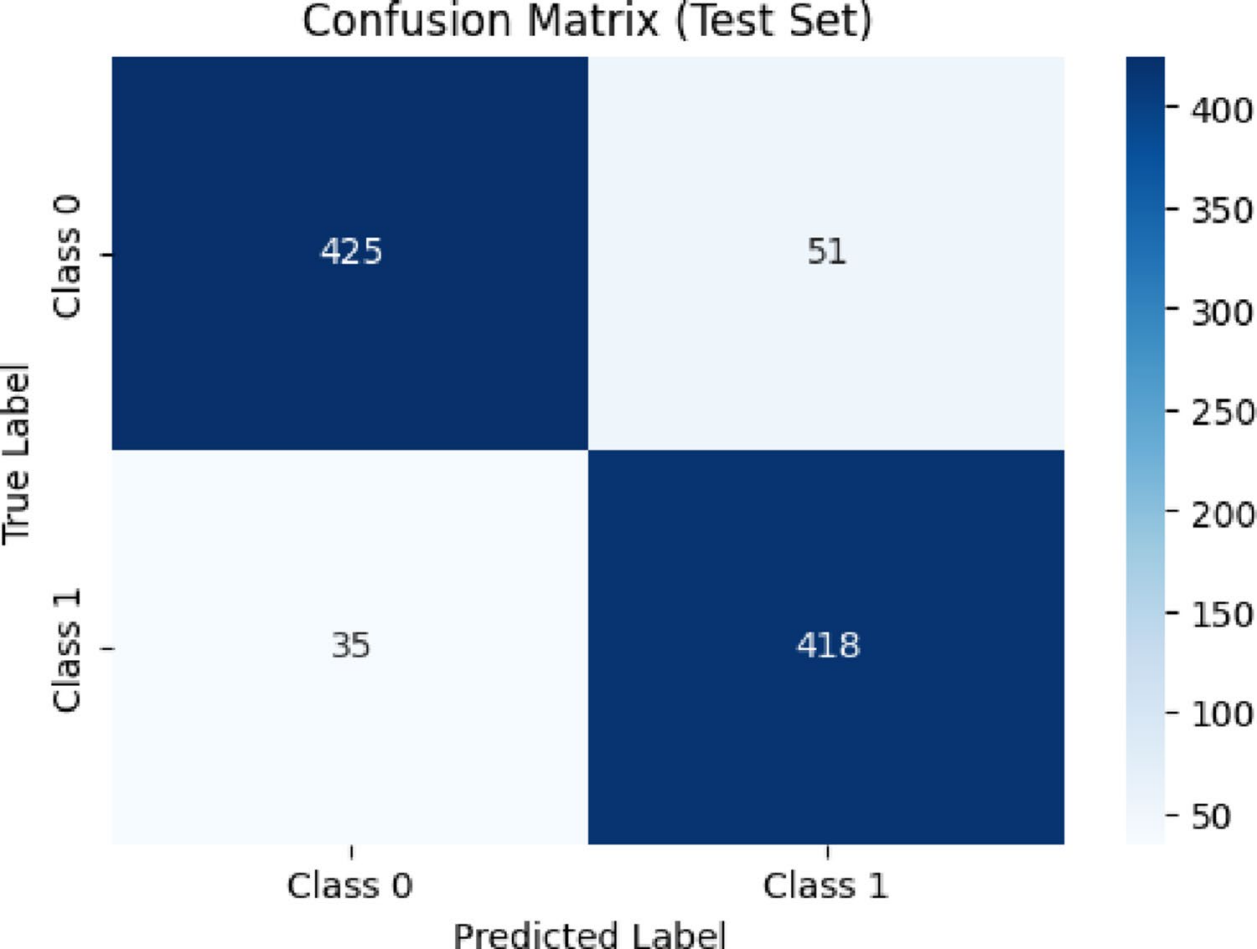
illustrates the confusion matrix of the XGBoost model for the test dataset

To assess external generalizability, the classifier was evaluated on the independent test set not involved in model training or hyperparameter optimization. The resulting confusion matrix (Fig. 3) demonstrated consistently strong predictive behavior, correctly identifying 418 active and 425 inactive molecules. Misclassifications consisted of 51 inactive compounds predicted as active (false positives) and 35 active compounds predicted as inactive (false negatives), corresponding to false-positive and false-negative rates of 10.7% and 7.7%, respectively. These error patterns are chemically plausible and align with FXa binding-site characteristics: borderline false positives commonly exhibit intermediate electrostatic or steric profiles resembling moderately active scaffolds, whereas false negatives often contain bulky or highly polar substituents that can interfere with optimal accommodation within the S1, S2, or S4 binding pockets [25].

The balanced distribution of false-positive and false-negative errors indicates that the classifier does not disproportionately favor either class, an essential property for virtual screening workflows. The combination of high test-set accuracy, strong ROC-AUC, and symmetric error patterns further confirms that the XGBoostClassifier effectively learns the nonlinear and high-order structure–activity relationships associated with FXa inhibition. These findings are consistent with recent computational studies demonstrating the utility of nonlinear machine-learning models, including ANN-QSAR frameworks, for capturing complex SAR patterns in FXa inhibitor datasets [26]. Collectively, the results support the suitability of XGBoost for early-stage drug-discovery applications such as hit prioritization, scaffold triaging, and activity enrichment.

### Applicability domain analysis

**Fig. 4:**
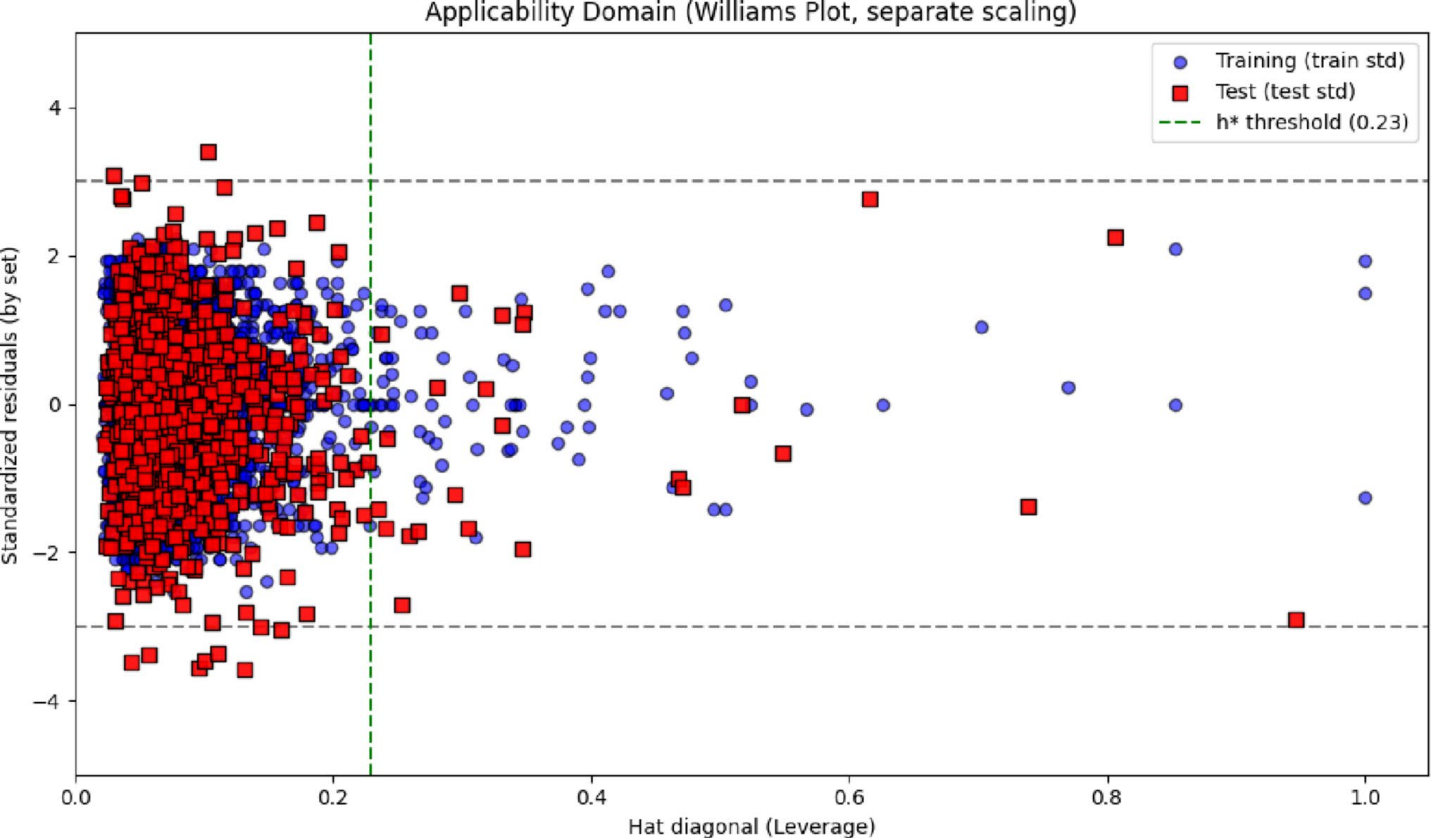
Williams plot representing the applicability domain (AD) of the regression model for both training and test sets

The reliability of the regression model across the explored chemical space was assessed using a Williams plot, which simultaneously displays the standardized residuals and leverage values for each molecule. As shown in Fig. 4, the vast majority of both training and test compounds fell within the predefined applicability domain (AD), bounded by the leverage threshold (h*) and standardized residual limits (± 3). Only a small number of molecules (77 in the training set and 35 in the test set) were identified as structural or statistical outliers. These compounds either exhibited leverage values exceeding h*, indicating an unusually high structural influence on the model, or showed large, standardized residuals consistent with atypical structure–activity relationships.

The presence of a limited fraction of outliers is expected in large and chemically diverse datasets such as FXa inhibitor libraries, which commonly include rare scaffolds, macrocyclic motifs, extended conjugation systems, or heavily functionalized heterocycles. Such structures may reside at the periphery of the chemical space and are naturally more difficult for the model to represent reliably. Importantly, the small proportion of outliers indicates that the descriptor–activity relationships learned by the model generalize well across the broader FXa inhibitor chemical landscape.

**Fig. 5:**
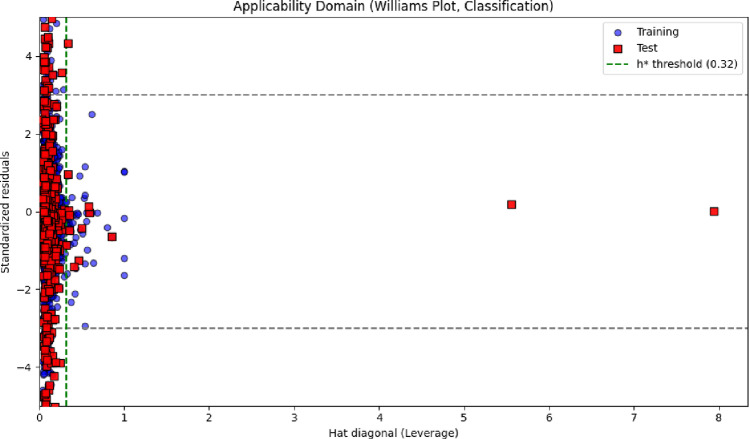
Williams plot of the classification model based on standardized residuals and leverage values

A similar applicability domain analysis was performed for the classification model to ensure consistency in prediction reliability. As shown in Fig. 5, most compounds in both the training and test sets were located within the AD boundaries, demonstrating broad domain coverage and stable model behavior across structurally diverse chemotypes. A total of 114 training molecules and 52 test molecules were flagged as outliers due to elevated leverage values or large standardized residuals, reflecting unusual structural features or atypical activity profiles.

These outliers typically corresponded to molecules with rare heteroaromatic frameworks, unusual charge distributions, or extended aromaticity—features that are underrepresented in the training set and therefore more challenging for the classifier to model accurately [28, 29]. Despite this, the relatively small number of outliers compared with the total dataset size confirms that the XGBoostClassifier maintains robust generalizability across a wide chemical space [30, 31].

While most predictions fall comfortably within the applicability domain, predictions associated with AD-flagged molecules should be interpreted cautiously, particularly in decision-critical tasks such as scaffold triaging, hit confirmation, or lead prioritization in virtual screening workflows. Integration of AD analysis therefore provides an essential safeguard, ensuring that high-confidence predictions are supported by sufficient structural representation in the training data [32–34]

### SHAP-based model interpretability

**Fig. 6:**
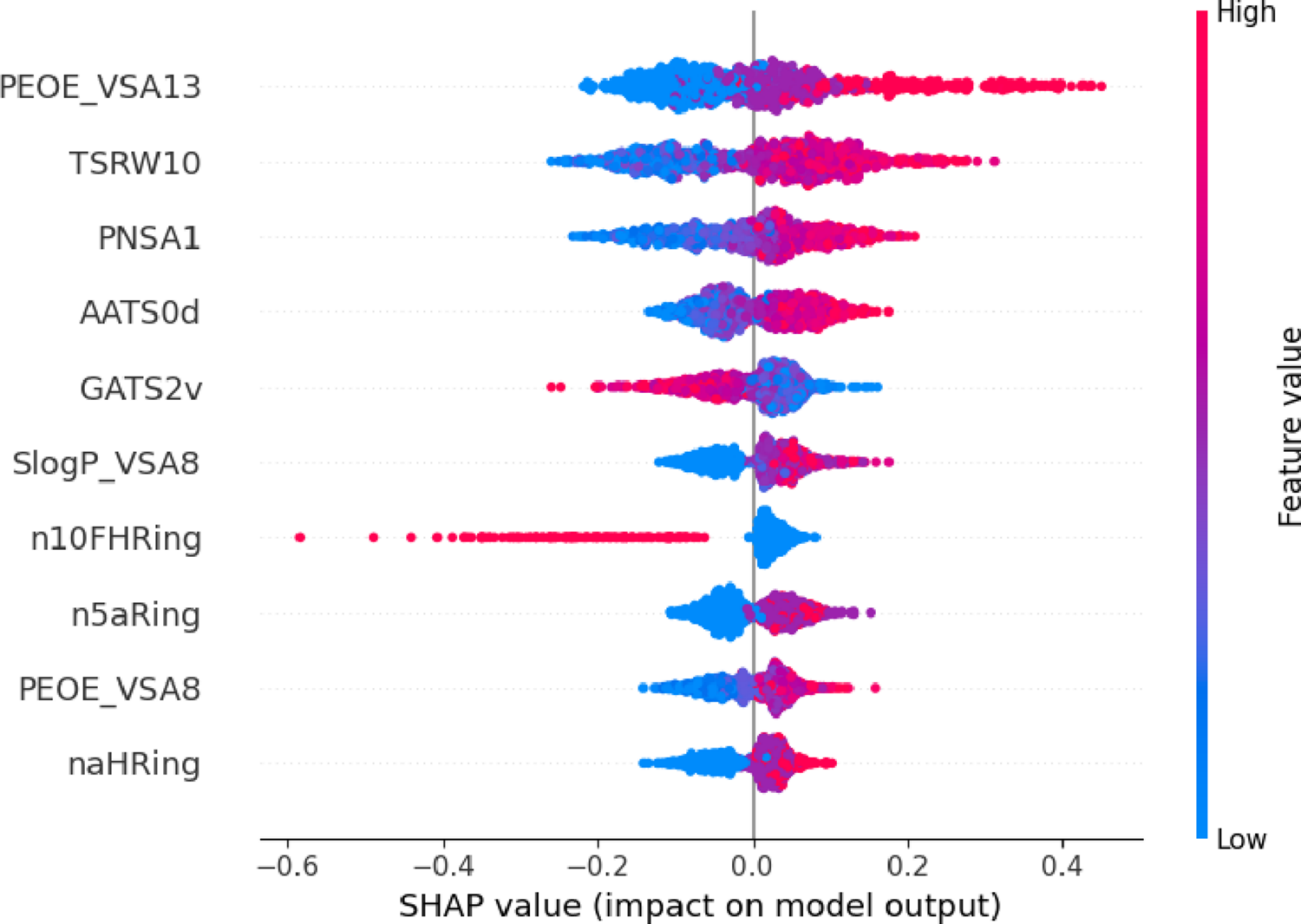
SHAP bar plot showing the top ten features with the highest average contributions to pKi predictions

To elucidate the molecular features governing FXa inhibitory potency, SHapley Additive exPlanations (SHAP) analysis was applied to the regression model to quantify the contribution of individual descriptors to pKi predictions. Figure 6 shows the ten most influential descriptors ranked by their mean absolute SHAP values. Among these, PEOE_VSA13, TSRW10, and PNSA1 displayed the highest contributions, indicating that electrostatic surface area distributions, molecular shape parameters, and polar surface characteristics play dominant roles in modulating FXa binding affinity.

The prominence of electrostatic descriptors such as PEOE_VSA13 and PNSA1 is consistent with the well-characterized FXa binding environment. Structurally, the FXa active site contains a deep, negatively charged S1 pocket, a shallow hydrophobic S2 region, and an aromatic–hydrophobic S4 subsite that collectively rely on electrostatic and steric complementarity to stabilize ligand interactions [25]. Accordingly, molecules exhibiting optimized charge distribution or enhanced polar surface exposure tend to form stronger ionic and hydrogen-bonding interactions, resulting in higher predicted potency.

Topological and shape-related descriptors, such as TSRW10, further underscore the importance of molecular geometry in achieving an optimal fit across the S1–S4 binding groove. This observation is consistent with crystallographic analyses suggesting that clinically used FXa inhibitors adopt elongated, shape-complementary conformations to establish multiple stabilizing interactions within these subpockets [25, 26].

**Fig. 7:**
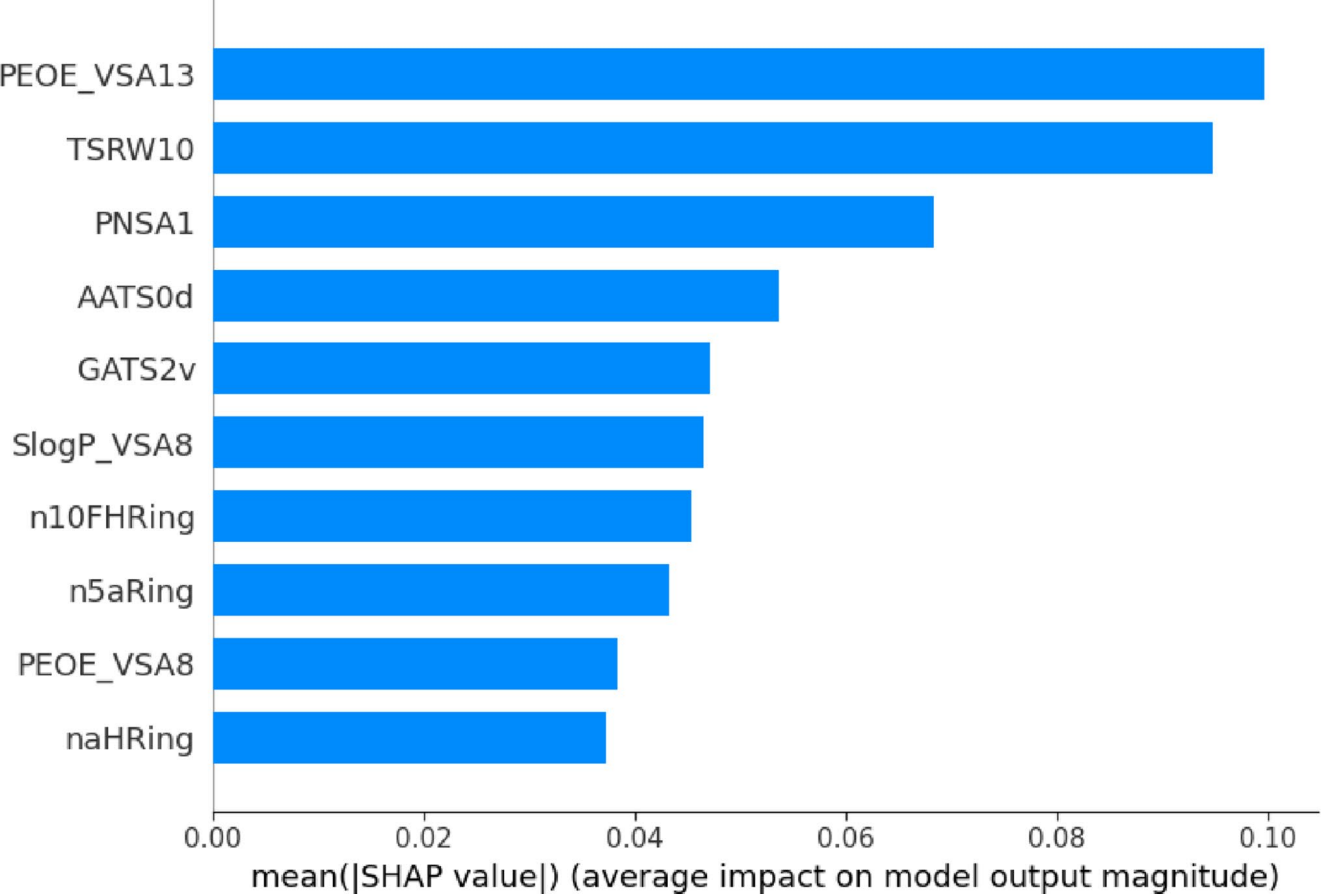
SHAP summary plot (bee swarm) displaying the distribution of SHAP values for the top ten features across the test sets

The SHAP summary plot (Fig. 7) provides a global view of the directionality and magnitude of descriptor effects. Higher PEOE_VSA13 values were consistently associated with increased predicted pKi, reaffirming the relevance of electrostatic complementarity. Conversely, low n10FHRing values—indicative of limited or absent heteroaromatic ring systems—correlated with reduced potency. This trend aligns with structure–activity studies showing that heteroaromatic frameworks facilitate π–π stacking, hydrogen bonding, and directional interactions essential for high-affinity FXa binding [26].

Descriptors such as AATS0d and GATS2v, which encode autocorrelation-based electronic and valence-related interactions, exhibited nonlinear patterns characteristic of complex SAR landscapes. Such behavior highlights why ensemble-based learning approaches outperform linear QSAR models when modeling multifactorial FXa inhibitor datasets [9].

Overall, the SHAP analysis demonstrates that the regression model captures chemically meaningful and mechanistically interpretable structure–activity relationships rather than relying on spurious correlations. These insights offer actionable guidance for future scaffold optimization aimed at enhancing FXa inhibitory potency.

## Conclusion

In this study, we developed a comprehensive, scalable, and interpretable machine-learning-based QSAR framework for predicting both the inhibitory potency and binary activity of small-molecule Factor Xa (FXa) inhibitors. By integrating a large and chemically diverse dataset with high-dimensional Mordred descriptors, benchmarking-driven model selection, and domain-aware validation, the proposed workflow demonstrated consistent predictive performance and reliable generalization across distinct chemical scaffolds.

The regression model built using the ExtraTreesRegressor achieved strong predictive accuracy (R^2^ = 0.760; RMSE = 0.831), indicating its ability to capture the nonlinear, multifactorial determinants of FXa binding affinity. Applicability domain analysis confirmed that most compounds fell within reliable prediction boundaries, while SHAP-based interpretability revealed that electrostatic, topological, and polar surface descriptors—including PEOE_VSA13, TSRW10, and PNSA1—play key mechanistic roles in shaping FXa inhibitory potency. These findings align with established pharmacophore characteristics of FXa ligands and provide valuable insights for future structure-based optimization. Likewise, the XGBoostClassifier yielded excellent classification performance (accuracy = 0.91; ROC-AUC = 0.962), with balanced precision–recall behavior underscoring its robustness in distinguishing active from inactive compounds.

Despite these strengths, the study has several limitations. First, the models rely exclusively on two-dimensional descriptors, excluding 3D conformational, stereochemical, and protein–ligand interaction information that may further refine FXa activity predictions. Second, variability in ChEMBL-reported bioactivity measurements may introduce noise during training. Third, the study relied solely on internal train–test splits, and no external validation dataset was used, which may limit the extrapolation of predictions to entirely novel chemical spaces. Finally, prediction reliability decreases near the boundaries of the applicability domain, warranting cautious interpretation for structurally atypical molecules.

Overall, the proposed QSAR pipeline offers a transparent, interpretable, and methodologically rigorous platform for virtual screening, scaffold hopping, and rational design of next-generation FXa inhibitors. Future work should incorporate external validation, prospective virtual screening, and structure-based methods such as molecular docking and molecular dynamics simulations to provide complementary insights. Ultimately, experimental evaluation of high-confidence predictions will be crucial for accelerating the discovery of safer, more selective, and clinically promising FXa inhibitors.

## Data Availability

The bioactivity data supporting the findings of this study were obtained from the ChEMBL database (Target ID: CHEMBL244). Processed datasets, molecular descriptors, and machine learning scripts used in this study are available from the corresponding author upon reasonable request.
